# MicroRNA and gene expression patterns in the differentiation of human embryonic stem cells

**DOI:** 10.1186/1479-5876-7-20

**Published:** 2009-03-23

**Authors:** Jiaqiang Ren, Ping Jin, Ena Wang, Francesco M Marincola, David F Stroncek

**Affiliations:** 1 Department of Transfusion Medicine, Clinical Center, National Institute of Health, 9000 Rockville Pike, Bethesda, Maryland 20892, USA

## Abstract

**Background:**

The unique features of human embryonic stem (hES) cells make them the best candidate resource for both cell replacement therapy and development research. However, the molecular mechanisms responsible for the simultaneous maintenance of their self-renewal properties and undifferentiated state remain unclear. Non-coding microRNAs (miRNA) which regulate mRNA cleavage and inhibit encoded protein translation exhibit temporal or tissue-specific expression patterns and they play an important role in development timing.

**Results:**

In this study, we analyzed miRNA and gene expression profiles among samples from 3 hES cell lines (H9, I6 and BG01v), differentiated embryoid bodies (EB) derived from H9 cells at different time points, and 5 adult cell types including Human Microvascular Endothelial Cells (HMVEC), Human Umbilical Vein Endothelial Cells (HUVEC), Umbilical Artery Smooth Muscle Cells (UASMC), Normal Human Astrocytes (NHA), and Lung Fibroblasts (LFB). This analysis rendered 104 miRNAs and 776 genes differentially expressed among the three cell types. Selected differentially expressed miRNAs and genes were further validated and confirmed by quantitative real-time-PCR (qRT-PCR). Especially, members of the miR-302 cluster on chromosome 4 and miR-520 cluster on chromosome 19 were highly expressed in undifferentiated hES cells. MiRNAs in these two clusters displayed similar expression levels. The members of these two clusters share a consensus 7-mer seed sequence and their targeted genes had overlapping functions. Among the targeted genes, genes with chromatin structure modification function are enriched suggesting a role in the maintenance of chromatin structure. We also found that the expression level of members of the two clusters, miR-520b and miR-302c, were negatively correlated with their targeted genes based on gene expression analysis

**Conclusion:**

We identified the expression patterns of miRNAs and gene transcripts in the undifferentiation of human embryonic stem cells; among the miRNAs that are highly expressed in undifferentiated embryonic stem cells, the miR-520 cluster may be closely involved in hES cell function and its relevance to chromatin structure warrants further study.

## Background

Human embryonic stem (hES) cells possess unique features: self-renewal and pluripotency. They can be continuously cultured in undifferentiated status and give rise to differentiated cells and tissues of all three germ layers. With these unique properties, it is reasonable to postulate that hES cells are the best resource not only for cell replacement therapy but also for studying human developmental biology. However, little has been done to understand the molecular mechanisms responsible for the maintenance of the undifferentiated status and the differentiation process of human embryonic stem cells.

MicroRNAs (miRNAs) are small (19 to 25 nts) endogenous non-coding RNA molecules that post-transcriptionally regulate gene expression [1,2]. Some miRNAs interact with their targets through imprecise base-pairing, resulting in the arrest of translation [3,4]; while others interact with their mRNA targets through near-perfect complementary and direct targeted mRNA degradation [5,6]. Many miRNAs exhibit temporal or tissue-specific expression patterns [7,8], and are involved in a variety of developmental and physiological processes [9,10].

It has been reported that miRNAs play an important role in mediating the regulation of development. For example, Dcr-1, which is essential for miRNA biogenesis, is required in germline stem cell (GSC) division in Drosophila melanogaster [11]; miR-143 regulates the differentiation of adipocytes [12]; miR-1 regulates cardiac morphogenesis, electrical conduction, and the cardiac cell cycle [13]; miR-181 is related to differentiation of B-lineage cells [14], while miR-155 is associated with development of immune system [15]. Signature miRNAs, such as the miR-302 family, the miR-200 family have been reported in human [16,17] and mouse embryonic stem cells [18-20]. The unique patterns of miRNA expression in embryonic stem cells suggest they are involved in maintaining "stemness".

Identifying mRNAs that are directly targeted by a specific miRNA is a major obstacle in understanding the miRNA functions. Computational prediction of miRNA targeted genes based on multiple parameters such as 5' seed sequence matching, free energy score and conservation among different species have been informative and rewarding, but lack experimental confirmation. Simultaneous profiling of miRNA and mRNA expression from the same sample can be a good strategy to identify functional miRNA targets in addition to computational selection. For miRNAs which lead to targeted mRNA degradation, their expression profile should reveal an inverse relationship with their cognate targets. A global analysis of both miRNAs and mRNAs expression across 16 human cell lines identified inverse correlated pairs of miRNA and mRNA [21]. Another analysis using 88 normal and cancerous tissue samples found that miRNA-mRNAs paired expression profiles could improve the accuracy of miRNA-target prediction on a large-scale [22]. However, the relationship between hES-specific miRNAs and their target genes is not yet well documented. To our knowledge there is only one article addressing this question, but it reported that negative correlations of miRNA and mRNA do not directly predict functional targeting in human embryonic stem cells [17].

In the present study, we applied two custom microarray platforms to detect global expression profiles of miRNAs and transcripts using three hES cell lines, embryonic bodies (EB) produced from one of the cell lines and five types of terminally differentiated adult cells. The integration of miRNA expression levels with gene expression levels provided evidence to support the negative correlation between hES-specific miRNAs and their target mRNAs expression level as a whole in human embryonic stem cells. These results will help to unravel the biological signalling pathways of hES cells.

## Results

### MiRNA expression profiling

The expression of hES-specific markers was assessed by immunofluorescence and flow cytometry. Our results revealed that over 90% of the hES cells were positive for Oct4, Nanog, Sox2, Tra-1-81, and Ssea4, but negative for Ssea1, suggesting that most of the hES cells were in an undifferentiation state.

**Figure 1:**
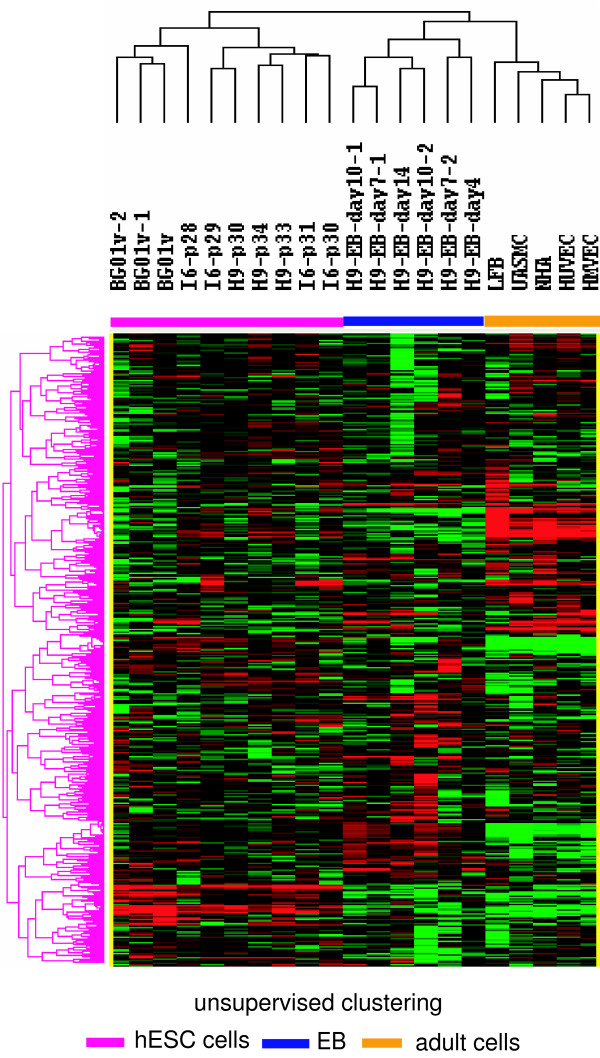
**Unsupervised hierarchical clustering of miRNAs**. The expression levels of miRNAs were presented as normalized cy5/cy3 ratios, upregulated miRNAs were shown as red and downregulated miRNAs were shown as green. I6, H9 and BG01v are names of human embryonic stem (hES) cells lines. P denoted the number of passages of the cell lines. H9-EB denoted embryoid body (EB) prepared from cell line H9 and the day indicates the time in culture. HMVEC = human microvascular endothelial cells, HUVEC = human umbilical vein endothelial cells, UASMC = umbilical artery smooth muscle cells; NHA = normal astrocyte and LFB = lung fibroblasts. Unsupervised hierarchical clustering analysis separated the samples to three major groups: hES cells, embryoid body (EB), and adult cells.

Global miRNA expression was analyzed among the 10 samples from 3 undifferentiated hES cell lines, 6 samples from EB and 5 samples from adult cell via a microarray platform (Gene Expression Omnibus accession number GSE12229). Unsupervised hierarchical clustering analysis separated the samples to three major groups: the hES cells, embryoid body (EB), and adult cells (Figure 1). Without statistic stratification, signature miRNAs specific for hES were distinguishable from EB and adults cell suggesting a diverse biological entity and fundamental difference in miRNA expression patterns.

**Figure 2:**
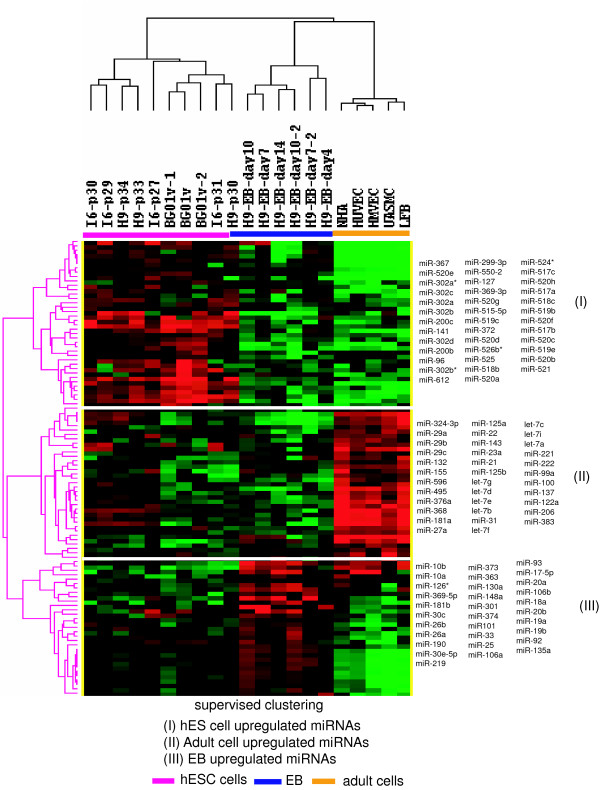
**supervised hierarchical clustering of miRNAs**. Supervised clustering using the 104 differentially expressed miRNAs classified the samples into three groups as well: hES, EB, and adult cells. Node I contained the miRNAs that were upregulated in hES cells, node II contained the miRNAs upregulated in adult cells, node III contained the miRNAs upregulated in EB. HMVEC = human microvascular endothelial cells, HUVEC = human umbilical vein endothelial cells, UASMC = umbilical artery smooth muscle cells; NHA = normal astrocyte and LFB = lung fibroblasts.

We identified 104 miRNA differentially expressed by the hES, EB and adult cell types (F-test, *P *< 0.01, FDR < 0.05). These included 38 miRNA upregulated in hES cells, 31 upregulated in EB cells, and 35 upregulated in adult cells (Figure 2). The 20 miRNAs most highly expressed in hES cells, EB, and adult cells respectively were shown in additional file 1. MiR-302a, miR-302b, miR-302c, miR-302d, miR-367, and miR-200c were increased in hES and have previously been reported to be hES-specific [16,17]. Moreover, the upregulation of these miRNAs in hES was confirmed by qRT-PCR with high correlation (R^2 ^= 0.65–0.9, data not shown).

Most miRNAs that are organized in clusters in close proximity on a chromosome have similar expression levels, indicating the possibility of transcribed in polycistronic fashion under the same promoter [16,23]. From our data, the expression of miR-302a, miR-302b, miR-302c, miR-302d and miR-367, which are co-located in a cluster on chromosome 4 were highly correlated (R^2 ^= 0.78–0.98). Likewise, miR-200c and miR-141 located in a cluster on chromosome 12 were also highly correlated (R^2 ^= 0.94). Our results also confirmed other miRNAs that are upregulated in hES cells such as miR-299-3p, miR-369-3p, miR-96 and miR-372[16,17,24,25]. However, miR-371, which is located in the same cluster with miR-372, was not discovered to be upregulated in hES cells by our results. Another member in this cluster, miR-373, was found to be upregulated in EB by our results, which was consistent with a recent report [26]. The differences among these studies may be attributed to the different cell lines tested or the different technical platforms used in assessing miRNA expression.

Most interestingly, 21 miRNAs located in a cluster on chromosome 19 exhibit similar expression levels. A portion of this large cluster has previously been found to be primate-specific and placenta-associated [27,28]. Among these miRNAs, miR-518b, miR-518c, miR-519b, miR-519c, miR-520a, miR-520c, miR-520e, miR-520g, and miR-524* are over-expressed in undifferentiated hES cells [24,26,29]. Besides these 9 miRNAs, we also identified 12 more miRNAs in this cluster; they were miR-515-5p, miR-517a, miR-517b, miR-517c, miR-519e, miR-520b, miR-520d, miR-520f, miR-520h, miR-521, miR-525-3p, and miR-526b*. The similar expression levels of these miRNAs imply that they may share functional similarity.

We identified three miRNA clusters that were upregulated in embryoid body (EB). One was the Oncomir cluster consisting of miR-17-5p, miR-20a, miR-18a, miR-19a, miR-19b, and miR-92a located on chromosome 13. The second was located on chromosome 7 and includes miR-25, miR-93 and miR-106b. The third was located on chromosome X and includes miR-106a, miR-363, and miR-20b. We also identified EB upregulated miRNAs that have not been previously reported such as miR-130a, miR-301a, and miR-135, miR-190, miR-30c, and miR-30e.

A maternally-expressed imprinted miRNA cluster on chromosome 14 [30] was upregulated in adult cells. This cluster included miR-495, miR-376a, and miR-369-5p. In addition, we identified 8 miRNAs of the let-7 family that upregulated in adult cells, whose expression was detected in hES cells [16,26], and was upregulated at the end of embryonic development [31].

### Gene expression profiling

**Figure 3:**
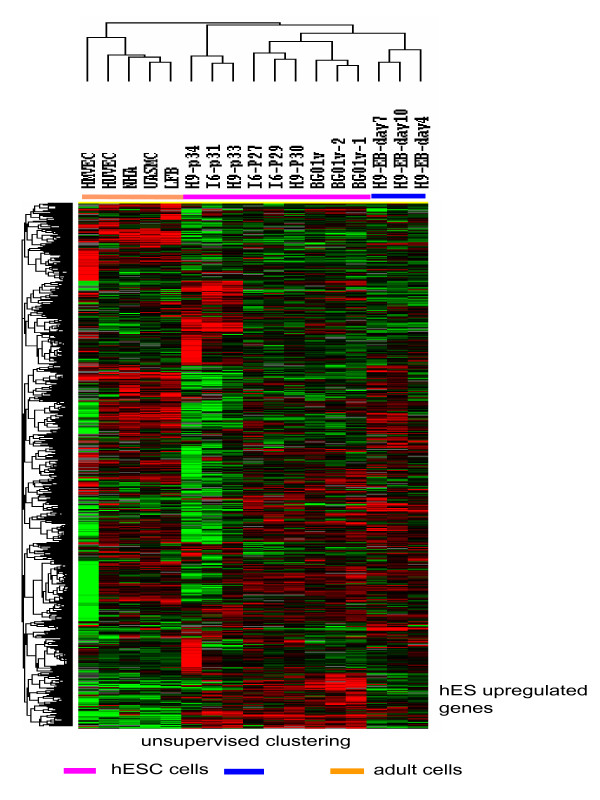
**unsupervised hierarchical clustering of genes**. The gene expression data is presented as normalized Log cy5/cy3 ratios, upregulated genes are shown as red, downregulated genes are shown as green. I6, H9 and BG01v are names of hES cells lines. P denotes the number of passages of the cell lines. H9-EB denotes embryoid body (EB) prepared from cell line H9 and the day indicates the time in culture. HMVEC = human microvascular endothelial cells, HUVEC = human umbilical vein endothelial cells, UASMC = umbilical artery smooth muscle cells; NHA = normal astrocyte and LFB = lung fibroblasts. Unsupervised hierarchical clustering analysis separated the samples to three major groups: hES cells, embryoid body (EB), and adult cells; the node containing hES markers was highlighted by white lines.

We assessed global hES gene expression profiles on 3 separate passages of cells from 3 different hES cell lines, EB samples at 3 different time points, and 5 types of adult cells, HUVEC, HMVEC, UASMC NHA, and LFB using a custom spotted oligonucleotide microarray (Gene Expression Omnibus accession number GSE12228). Unsupervised hierarchical clustering using filtered genes classified the samples into three groups: the hES group, EB group and adult cell group. This clustering analysis also identified one node containing the hES cell markers *POU5F1 (OCT4), LEFTY1, TDGF1 *and *DPPA4 *(Figure 3).

**Figure 4:**
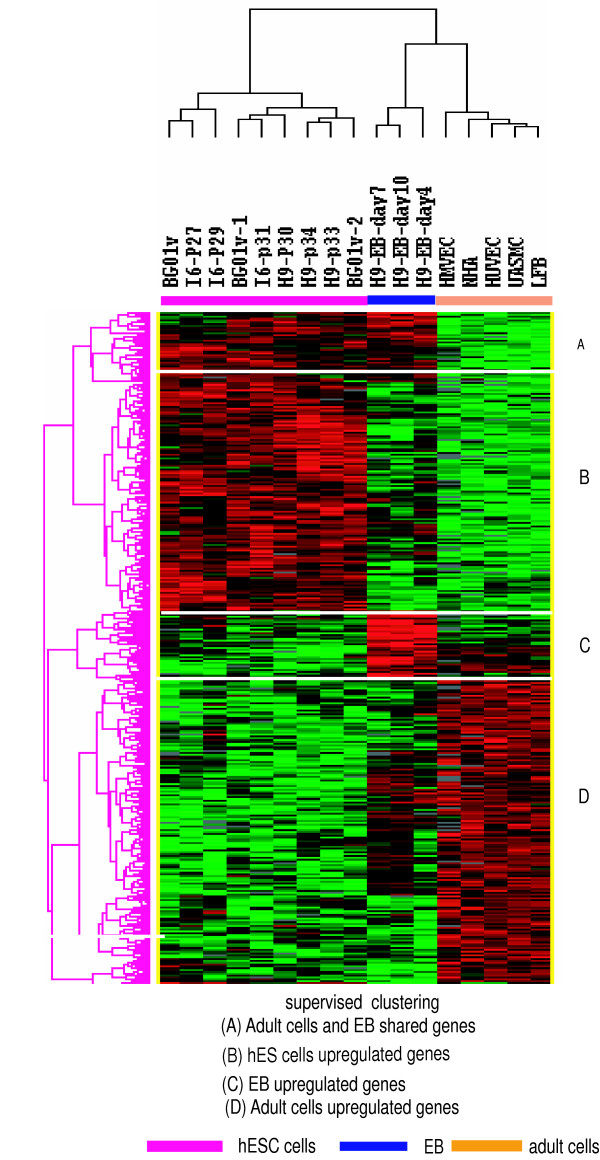
**supervised hierarchical clustering of genes**. Supervised clustering using the differentially expressed gene classified the samples into three groups: hES cells, EB, and adult cells. Node A contained the genes that were upregulated in both hES cells and EB, node B contained the genes upregulated in hES cells only, node C contained the genes upregulated in EB only, and node D contained the genes that were upregulated in adult cells. HMVEC = human microvascular endothelial cells, HUVEC = human umbilical vein endothelial cells, UASMC = umbilical artery smooth muscle cells; NHA = normal astrocyte and LFB = lung fibroblasts.

We identified 776 genes differentially expressed among hES, EB and adult cell types (F-test, cut-off *p *< 0.005, FDR < 0.05). Hierarchical clustering analysis of these genes also divided the samples into three groups, hES, EB, and adult cells, and divided the genes into 4 major nodes (Figure 4). The node containing 226 genes that were upregulated in hES cells (node B) included the previously identified hES markers *OCT4, TDGF1, LEFTY1, DNMT3B, GAL, DPPA4, UGP2, TERF1, GABRB3, CD24, FAM46B, SALL4, TCEA1, ZNF398, NODAL, and ACVR2B *[32-35]. The node containing genes upregulated in EB (node C) included the genes *HAND1, HOXA1, HOXB2, MSX1, MSX2, MEIS1, FGF9 *and *FREM1 *which are involved in morphogenesis and development [36-39]. This node also included transcription factors *GATA5, ELF3, MSRB2, MIER1, XRCC6 *and *ZFHX3 *which are related to development. A node containing a small number of genes that were upregulated both in EB and in hES cells (node A) included *GLI1, ISL1, CRABP1*, and *KRT9*. Of note is that *GLI1 *activation is required in sonic hedgehog (Shh) signalling pathway [40], which is essential in regulating development, stimulation of the Shh pathway also results in the upregulation of *GLI1 *in hES cells [41], suggesting that *Gli1 *plays an important role in embryological development and hES cell differentiation.

### Correlation of miRNAs and their predicted targets

**Figure 5:**
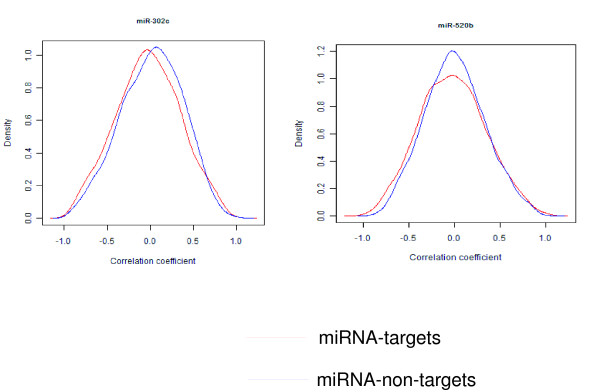
**Correlation coefficients of miRNA-target gene pairs**. The expression of miR-302c and miR-502b and their predicted target genes was analyzed by correlation analysis. The distribution of the correlation coefficients for miR-302c-target gene pairs (red line) was shifted toward negative side compared to that of the miR-302c-non-target gene pairs (blue line). The mean of correlation coefficients between the two sets was significantly different (*p *= 0.0003). The distribution of correlation coefficients for miR-520b-target gene pairs (red line) was also shifted toward negative side compared to the miR-520b-non-target gene pairs (blue line) and the mean of correlation coefficients was significant (*p *= 0.049).

The mRNAs that are predicted to be targets of specific miRNAs are expressed at significantly lower levels [42,43]. This is likely caused by miRNA-mediated destabilization of target mRNA. To determine whether the negative correlation between miRNA and gene expression levels actually reflected miRNA-target relationships in hES cells, we calculated the correlation coefficients between the expression levels of hES upregulated miRNAs and the levels of their predicted targets. The predicted targets for each miRNA were downloaded from miRNAMap2.0 [44] and their expression value were extracted from our gene expression microarray data. To avoid random correlation, we calculated the correlation coefficients between miRNA expression levels and randomly-selected non-target genes of the same number. In general, the expression levels of miRNAs were both positively and negatively correlated with their predicted targets for all the miRNAs analyzed. However, we still observed a preponderance of negative correlation over positive correlation between some specific miRNAs and their targets. The distribution of the correlation coefficients for miR-302c-target genes was shifted toward the negative side compared to that of the miR-302c-non-target genes. This was also true for the miR-520b-target genes. The mean of the correlation coefficients between the two sets, targeted and non-targeted genes, was significantly different (*p *= 0.0003 for miR-302c and *p *= 0.049 for miR-520b) (Figure 5).

### Validation of microarray results

**Figure 6:**
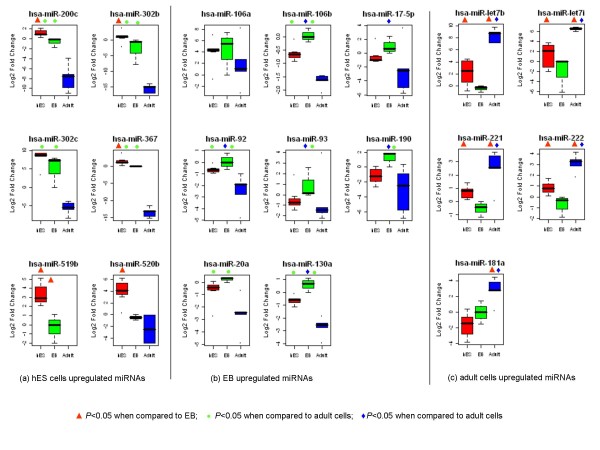
**Measurement of differentially expressed miRNAs by qRT-PCR**. The differentially expressed miRNAs were analyzed by qRT-PCR using the relative quantification method. The results were normalized with endogenous control RNU48 and the fold change was calculated by equation2^-ΔΔCt^. The y-axis indicates the Log2-transformed fold change relative to the calibrator. Expression of levels of miR-200c, miR-302b, miR-302c, miR-367, miR-519b, and miR-520b were the greatest in hES cells (panel A). The expression of miR-106a, miR-106b, miR-17-5p, miR-92, miR-93, miR-190, miR-20a and miR-130 were highest in EB (panel B). Tumor suppressor let-7b/7i, miR-221, miR-222 and miR-181a were expressed at the highest levels in adult cells (panel C). Statistical significance was determined by student t-test. Red triangles indicate a significant difference (*P *< 0.05) versus EB, green circles indicate a significant difference (*P *< 0.05) versus adult cells, and blue diamonds indicate a significant difference (P < 0.05) versus hES cells.

Using qRT-PCR we found that the expression levels of miR-302b, miR-302c, miR-367, miR-200c, miR-519b, and miR-520b were much higher in hES cells than in either EB or adult cells (Figure 6, panel A). The difference in the expression of miR-200c, miR-302b, and miR-367 between hES cells and EB, and between hES cells and adult cells was significant (*P *< 0.05). The difference in miR-302c expression between hES cells and adult cells was also significant (*P *< 0.05). In particular, the expression of miR-519b was 8-fold greater in hES cells than in EB cells and it was not even detected in adult cells. The expression of miR-520b was 26-fold greater in hES cells then in EB cells (*P *< 0.05) and it was detected only in two types of adult cells HMVEC and HUVEC.

Differences in the expression of EB signature miRNA were also confirmed by qRT-PCR. The expression of miR-106a, miR-106b, miR-17-5p, miR-92, miR-93, miR-130a, miR-20a and miR-190 were much higher in EB than in either hES cells or adult cells (Figure 6, panel B). For miR-106b, miR-92, miR-93, miR-130a and miR-190, the difference in their expression between EB and hES cells and between EB and adult cells were significant (*P *< 0.05). The difference in expression of miR-17-5p between EB and hES cells, and of miR-20a between EB and adult cells were also significant (*P *< 0.05).

We also confirmed that let-7b, let-7i, miR-221, miR-222 and miR-181a were much more highly expressed in adult cells (Figure 6, panel C). The differences in expression of these miRNA expression between adult cells and hES cells and between adult cells and EB were significant (*P *< 0.05). Of note, the expression levels of let-7b and let-7i were much higher in hES cells than in EB, and this result was consistent with both our microarray results and a previous report [26], indicating that the let-7 family plays important role in the maintaining hES cells function although their expression level was much lower than in adult cells. We also confirmed that miR-222 was more highly expressed in adult cells, although it was reported to be enriched in hES cells [17]. Actually, miR-222 was also expressed in multiple adult cell lines [16], forebrain and midbrain [45], and hippocampus [46]; it was upregulated in the differentiation process of undifferentiated hES cells to neural progenitor cells and then declined upon further differentiation [25]; it was also downregulated in erythropoietic culture of cord blood CD34+ progenitor cells [47].

**Figure 7:**
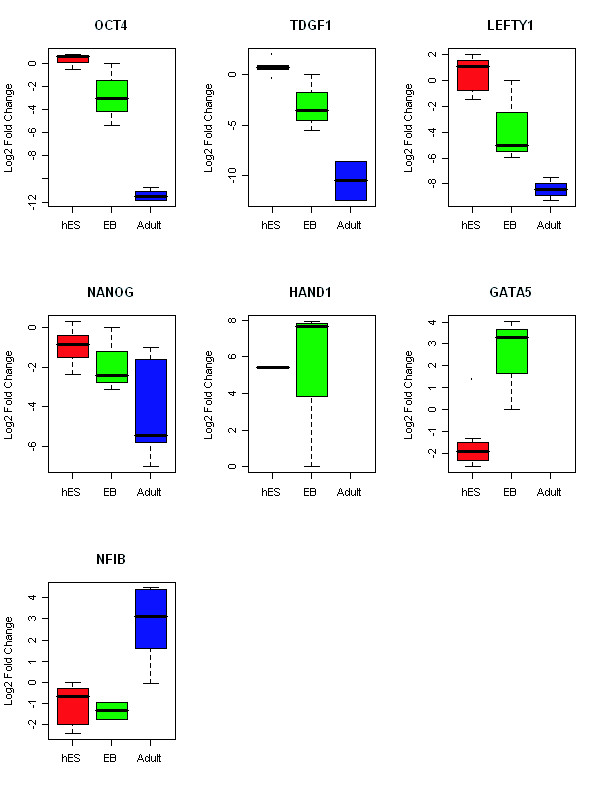
**Measurement of differentially expressed genes byqRT-PCR**. Quantitative real-time PCR confirmed the expression of 3 genes found by microarray analysis to be upregulated in hES: *POU5F1 (OCT4), LEFTY1*, and *TDGF1*, and 2 genes upregulated in EB: *HAND1 *and *GATA5*, and 1 gene upregulated in adult cells: *NFIB*. In addition, the levels of another hES cell marker *Nanog *was also measured. The results were normalized by endogenous control 18s rRNA and the fold change was calculated by equation2^-ΔΔCt^. The y-axis indicates the Log2-transformed fold change relative to the calibrator.

Selected differentially expressed genes identified by microarray analysis were also validated via qRT-PCR. Markers for hES cells, *POU5F1 (OCT4), LEFTY1 *and *TDGF1 *were highly expressed in hES cells (Figure 7). The expression of *OCT4 *by hES cells was upregulated by 12-fold, *LEFTY1 *by 70-fold and *TDGF1 *by 19-fold compared to EB. Compared to adult cells, expression of *OCT4 *in hES was increased by 4,324-fold, *LEFTY1 *by 769-fold, and *TDGF1 *by 2,443-fold. We did not find that *Nanog *was upregulated in hES cells by microarray analysis, and by qRT-PCR its expression was increased by only 3-fold in hES cells compared to EB cells and 25-fold to adult cells. This discrepancy may have resulted from the fact that microarray platform is less sensitive than qRT-PCR. Analysis by qRT-PCR confirmed that both *HAND1 *and *GATA5 *were upregulated in EB, but were not detected in adult cells; *HAND1 *was only expressed in 1 hES sample, *GATA5 *expression was increased by 37-fold in EB cells compared to hES cells. The expression level of *NFIB *was much higher in adult cells than in either hES cells or EB (Figure 7) which was also consistent with the microarray results.

### Functional comparison of miR-302 cluster and miR-520 cluster

**Figure 8:**
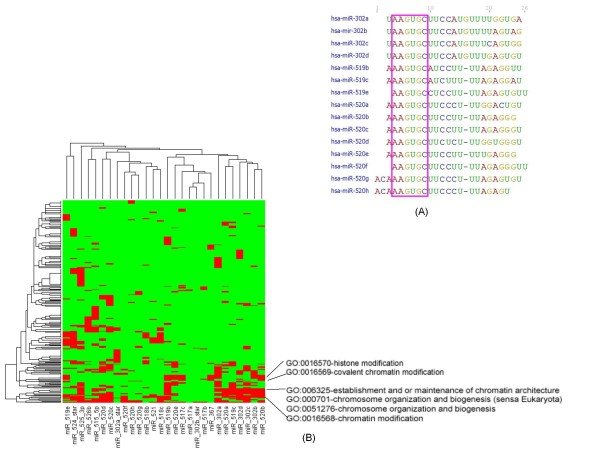
**Sequence and GO analysis of the miR-302 cluster and miR-520 cluster**. The members of the miR-302 and miR-520 clusters had similar sequences; they shared a consensus seed sequence: AAGUGC (panel A, seed sequence is highlighted by the purple rectangle). At the Gene Ontology level, miR-520b, miR-302b, miR-302c, miR-302d, miR-519c, miR-520a, and miR-302a formed a cluster (significant GO terms shown as red), and they shared GO terms related to chromatin structure modifications (Panel B).

Among the miRNAs upregulated in hES cells, we observed 7 miRNAs were located in the miR-302 cluster and 21 miRNAs were located in miR-520 cluster. Most of these miRNAs had highly similar sequences at the 5' end seed region. In particular, miR-302a, miR-302b, miR-302c, miR-302d, miR-519b, miR-519c, miR-520a, miR-520b, miR-520c, miR-520d, and miR-520e had a consensus seed sequence: AAGUGC (Figure 8, panel A). To infer the function of these miRNAs, we predicted 2,436 targets for the miR-302 cluster and 4,691 targets for the miR-520 cluster by querying the public database miRNAMap 2.0 , and 2,284 target genes were shared by both clusters suggesting functional similarity. Gene Ontology (GO) enrichment analysis confirmed that the inferred functions of miRNAs within the miR-302 and miR-520 clusters were overlapping based on their involvement in cell growth, negative regulation of cellular metabolic process, negative regulation of transcription, and small GTPase mediated signal transduction. To visualize the functions of these miRNA targeted genes, a binary (red indicate participate in the functional category and green indicate not) heatmap was used to indicate functional commonality among all miRNAs in miR-302 and miR-520 clusters. MiR-520b, miR-302b, miR-302c, miR-302d, miR-519c, miR-520a and miR-302a were clustered closely base on the 48 GO terms analyzed. Interestingly, out of 48 functional categories analyzed, 6 related to chromatin structure were identified in this cluster, which included histone modification, covalent chromatin modification, establishment and or maintenance of chromatin architecture, chromosome organization and biogenesis, and chromatin modification (Figure 8, panel B).

## Discussion

The present study investigated hES cell specific miRNAs profiles and transcription profiles through the comparison of partially differentiated EB and terminal differentiated adult cells. From miRNA array analysis, we identified a total of 104 differently expressed miRNAs that clearly segregate the three cell types analyzed. miRNAs expressed at high levels in hES cells and downregulated during differentiation or in adult cells included the well-known miR-302 family, miR-200 family, and miR-372. In addition, we identified 21 hES upregulated miRNAs that were co-localized in a cluster on chromosome 19, the miR-520 cluster, many of which shared consensus seed sequence with miR-302 family and which can be used as candidate biomarkers for pluripotency (Additional file 1).

In the present study, miR-200b, miR-200c and miR-141, all members of the miR-200 family, were upregulated in hES cells. The function of miR-200 family in hES is not well documented. It has been reported that miR-200 family targets E-cadherin transcriptional repressors ZEB1 and ZEB2, thus inhibiting epithelial to mesenchymal transition (EMT) [48-50], which facilitates tissue remodelling during embryonic development. The miR-200 family is also required for the proper differentiation of olfactory progenitor cells in zebrafish model [51], indicating that the miR-200 family is involved in development. It has been shown that the inhibition of miR-141 decreases growth of cholangiocarcinoma cells [52]. Moreover, miR-200 family have been reported to be upregulated in many malignant tumors such as hepatocellular carcinoma [53], malignant cholangiocytes [52], and ovarian cancer [54]. Thus our results are consistent with the previous report that oncogenic miRNAs were upregulated in hES cells[24], suggesting a possible function of blockade of cell differentiation.

Our results confirmed the recent report that majority of miRNA genes in hES cells were expressed from Chromosomes 19 and X [55] and demonstrated the significant upregulation of miR-520 cluster in hES cells. Less is known about the function of the miR-520 cluster. miR-520h has been reported to be highly expressed in hematopoietic stem cells (HSCs) from human umbilical cord blood, and it promotes differentiation of HSCs into progenitor cells by inhibiting *ABCG2 *expression[56].

Along with the reports of miR-302 family on chromosome 4 [16,17,19,25,26], several groups have reported the expression of members of miR-520 cluster on chromosome 19 in hES cells [24,26,29]. Nine of these miRNAs were consistent with our results. In addition, we identified 12 other hES upregulated miRNAs in this cluster: miR-302a, miR-302b, miR-302c, miR-302d, miR-519b, miR-519c, miR-520a, miR-520b, miR-520c, miR-520d, miR-520e which share a consensus seed sequence: AAGUGC [24]. The miR-302 cluster and miR-520 cluster target large groups of genes which share overlapping functions based on Gene Ontology (GO) analysis. The functions shared by these two clusters included cell growth arrest, negative regulation of cellular metabolic process, negative regulation of transcription, and small GTPase mediated signal transduction. These gene functions correlate with hES cells characteristics and biology suggesting a well controlled and maintained stability. Of special note is that predicted target genes for both clusters were associated with modification of chromatin structure, which plays essential roles in transcription regulation, DNA replication, DNA damage repair and cell cycle control. Embryonic stem cells have a unique bivalent chromatin structure which silences developmental genes in ES cells while keeping them poised for activation, thus providing a mechanism for maintaining pluripotency [57]. The upregulation of miR-302 cluster and miR-520 cluster in hES cells suggests their ability to modulate local chromatin states which is necessary for stem cell pluripotency [58,59].

Many of these miRNAs that were highly expressed in EB belong to the miR-17-92 cluster located on chromosome 13. The expression of miR-92 has been reported in human embryonic stem (ES) cells [16,26], mouse ES cells[20] or human EB [17] depending on the reference sample used for comparison. It should not be forgotten that hES cells contain spontaneously differentiated cells, so it is difficult to precisely determine which type of cells express miR-92. The members of miR-17-92 cluster and its paralogs such as miR-106a, miR-106b, miR-93, and miR-17-5p are related to DNA replication and cell mitosis in cancer cells [60-62], moreover, miR-17-5p and miR-20a can induce heterochromatic features in promoters that undergo overlapping transcription and possess sequence complementarity to the miRNA seed region [63]. The most important role of miR-17-92 cluster has been documented in association with oncogenic process, lymphoproliferative disorders, autoimmune disease and development [64-66]. Loss-of-function of the miR-17-92 cluster resulted in smaller embryos and immediate postnatal death of animals [67], which could due to the deficiency of their roles in the development of the heart, lung, and immune system [66]. Additionally, we discovered that miR-30c and miR-30e were upregulated in EB, which are expressed in human leukaemia cells [68], indicating that they have a role in controlling cell cycle and cell proliferation. This is in line with an analysis which revealed that EB-enriched miRNA targeted genes are involved in cell proliferation and is in contrast with the function of hES-enriched miRNAs targeted genes [26].

The miRNAs that were upregulated in adult cells included several members of the tumor suppressor let-7 family, which inhibits cell growth and tumor cells motility [31]. They are expressed in the brain [17,46], osteocytes [69], benign breast epithelial cells [61] and are downregulated upon malignant transformation [60,61,70]. Let-7 miRNAs also regulate late embryonic development by suppressing the expression of *c-myc*, RAS and high mobility group A2 (*HMGA2*) [19,71]. Recently, it was reported that the downregulation of let-7 is essential for self-renewal and maintenance of the undifferentiated state of cancer stem cells [72], indicating that this family of miRNAs has a greater role in stem cell function than previously described.

The currently available miRNA target prediction algorithms always result in high false-positive rates. Several reports have assumed that a negative correlation between miRNA and gene expression levels is an indicator for a miRNA-target gene relationship [21,43], if the function of the miRNA is dominant in leading the mRNA target degradation, however, most animal miRNAs pair to the 3' UTRs of their targets by incomplete base-pairing through their seed region [42]. We used the genome-wide miRNA and mRNA expression data for the global correlation analysis between miRNAs and their predicted target genes. As expected, both positive and negative correlations between hES-specific miRNAs and their targets were observed. The positive correlation indicates that the miRNAs were co-expressed with their targets, and it is tempting to speculate that miRNAs might function by suppressing the encoded protein translation of their targets rather than by leading mRNA cleavage. This positive correlation could also be due to other miRNA regulatory function. For instance, miR-373 induces the expression of *E-cadherin *and *CSDC2 *by targeting their promoter region and initiate their expression[73]. Another mechanism is that the engagement of miRNA and their targets at 3'UTR can sometimes stabilize the mRNA and prolong the encoded protein translation as exemplified by miR-155 which increases the translation of *TNF-α *[74].

As more experimental data has been accumulated, the versatile and complicated regulatory function of miRNA to their targets has become more apparent. To understand the predominant function of differentially expressed miRNA in the current study, we focused on miR-302c and miR-520b which were upregulated exclusively in hES and their correlation with computational predicted targeted genes. Although both upregulation and downregulation was observed among the targets, a greater portion of inverse correlation coefficients were detected between miRNA and their targets than non-target pairs suggesting a non-random correlation and possible miRNA induced mRNA cleavage function. This analysis can provide useful information concerning miRNA and their function in hES cell biology. For example, the expression of nuclear factor I/B (*NFIB*), one of miR-302c targeted genes, was repressed in hES cells and upregulated in EB and adult cells. *NFIB *is a transcription factor involved in brain development [75-78], chondrocytic differentiation [79] and lung development [78]. It is reasonable to assume that *NFIB *downregulation in hES may be involved in regulating hES pluripotency and undifferentiated status. Experiments are underway to test the function of miR-302c-target pairs.

## Conclusion

In the present study, we analyzed miRNA profiles and transcription profiles simultaneously on undifferentiated hES cell, partially differentiated EB cell and terminal differentiated cells and identified signature miRNA along with a specific gene signature for hES cells. The differentially expressed hES miRNAs were organized in clusters and their expression was negatively correlated with their predicted targets. Among the hES signature miRNAs, the miR-520 cluster shared a similar expression pattern and seed sequence as the well known miR-302 family and targeted the same genes as the miR-302 family. In addition to the inferred function of these miRNA in controlling cell growth, negative regulation of cellular metabolic process, negative regulation of transcription, and small GTPase mediated signal transduction; these two clusters have a similar inferred function in modification of chromatin structure.

## Methods

### Cell culture and embryoid body differentiation

Human embryonic stem cell lines WA09 (H9), TE06 (I6), and BG01v from WiCell Research Institute (Madison, WI), Technion-Israel Institute of Technology (Haifa, Israel) and ATCC (Manassas, VA) were cultured on mitotically inactivated mouse embryonic fibroblast (MEF) feeders using DMEM/F12 medium optimized for human ESC culture (GlobalStem Inc, Rockville, MD) supplemented with 20% knockout serum replacement and 4 ng/ml bFGF (both from Invitrogen, Gaithersburg, MD). Culture medium was changed daily and subculturing was performed every 4–6 days by collagenase IV (1 mg/ml) (Invitrogen, Gaithersburg, MD) digestion and mechanical disruption. The undifferentiation state of hES cells was determined by immunofluorescence detection of Pou5f1 (Oct4), Ssea4 (Millipore, Billerica, MA), Nanog (BD Bioscience, San Jose, CA), Sox2 (R&D Systems Inc. Minneapolis, MN), Tra-1-81 (Abcam, Cambridge, MA) and negative marker Ssea1 (Abcam, Cambridge, MA). The percentage of hES cells positive for Pou5f1 (Oct4), Sox2 and Ssea4 was measured by flow cytometry (FCM).

For embryoid body (EB) differentiation, hES cells were detached with collagenase IV and the cell aggregates were briefly triturated then cultured in ultra low attachment plates (Corning Inc, Corning, NY) for up to 14 days in maintenance medium. The medium was changed every three days.

The tested adult cells were Human Microvascular Endothelial Cells (HMVEC), Human Umbilical Vein Endothelial Cells (HUVEC), Umbilical Artery Smooth Muscle Cells (UASMC), Normal human astrocytes (NHA) (all from Lonza Inc, Walkersville, MD), and Lung Fibroblasts (LFB) (ATCC). All of the adult cells were cultured according to manufacturer's protocol.

### MiRNAs expression profiling

A miRNA probe set was designed using mature antisense miRNA sequences (Sanger data base, version 9.1) consisting of 827 unique miRNAs from human, mouse, rat and virus plus two control probes. The probes were 5' amine modified and printed in duplicate on CodeLink activated slides (General Electric, GE Health, NJ, USA) via covalent bonding at the Infectious Disease and Immunogenetics Section of the Department of Transfusion Medicine (DTM) (Clinical Center, NIH, Bethesda, MD). 4 μg total RNA isolated by using Trizol reagent (Invitrogen, Gaithersburg, MD) was directly labelled with miRCURY™ LNA Array Power Labelling Kit (Exiqon, Woburn, MA) according to manufacture's procedure. The total RNA from Epstein-Barr virus (EBV)-transformed lymphoblastoid cell line was used as the reference for the miRNA expression array assay. The test sample was labelled with Hy5 and the reference with Hy3. After labelling, the sample and the reference were co-hybridized to the miRNA array at room temperature overnight in the present of blocking reagents as previously described[80] and the slides were washed and scanned by GenePix scanner Pro 4.0 (Axon, Sunnyvale, CA, USA).

### Gene expression profiling

Total RNA was extracted using Trizol reagent and the RNA quality was tested with the Agilent Bioanalyzer 2000 (Agilent Technologies, Santa Clara, CA). The RNA was amplified into antisense RNA (aRNA) as previously described[80]. Total RNA from PBMCs pooled from six normal donors was extracted and amplified into aRNA to serve as the reference. Both reference and test aRNA were directly labelled using ULS aRNA Fluorescent Labelling kit (Kreatech, Salt Lake City, UT) with Cy3 for reference and Cy5 for test samples. Whole-genome human 36K oligo arrays were printed in the Infectious Disease and Immunogenetics Section of Transfusion Medicine, Clinical Center, NIH (Bethesda, MD) using a commercial probe set which contains 35,035 oligonucleotide probes, representing approximately 25,100 unique genes and 39,600 transcripts excluding control oligonucleotides (Operon Human Genome Array-Ready Oligo Set version 4.0, Huntsville, AL). The design is based on the Ensemble Human Database build (NCBI-35c), with a full coverage on NCBI human Refseq dataset (04/04/2005). Hybridization was carried out at 42°C for 18 to 24 hours and the arrays were then washed and scanned on a GenePix scanner Pro 4.0 at variable photomultiplier tube to obtain optimized signal intensities with minimum (< 1% spots) intensity saturation.

### Microarray data analysis

The resulting gene expression data files were uploaded to the mAdb database and further analyzed using BRBArrayTools developed by the Biometric Research Branch, National Cancer Institute . Briefly, the raw data set was filtered according to standard procedure to exclude spots with minimum intensity and size. Then, the filtered data were normalized using Lowess Smoother. For miRNA array, the signal intensities were extracted via the R programming language (version 2.6.0, ) and the libraries provided by the Bioconductor project[81]. The background-subtracted data were then subject to variance stabilization normalization[82] and imported into BRBArray Tools . Differentially expressed miRNAs or genes were identified using F-tests with a *P*-value cutoff of 0.01 (miRNA) or 0.005 (gene); *P*-values were adjusted for multiple comparisons by False Discovery Rate < 0.05. Clustering and visualization of expression profiles was preformed with Cluster and Treeview software [83]. The correlation between miRNA and target genes was performed using CCA package [63]; for comparison, the expression level of non-target genes of the same number was also correlated with the miRNA expression. Density plot of correlation coefficient distribution was generated in R environment.

### Validation of differentially expressed genes and miRNAs by qRT-PCR

For validation of microarray data, differentially expressed genes were detected by using the pre-designed TaqMan^® ^Gene Expression Assays (Applied Biosystems, Foster City, CA). Differentially expressed miRNAs were measured by TaqMan microRNA Assays as previously reported [84]. The differences of expression were determined by relative quantification method; the Ct values of the test genes or miRNAs were normalized to the Ct values of endogenous control (RNU48 for miRNA and 18s rRNA for mRNA). The fold change was calculated using the equation 2^-ΔΔCt^.

### Gene target prediction for miRNAs and Gene Ontology (GO) analysis

Gene target prediction was performed by querying the miRNA Database miRANDA [85] and RNAhybrid [86] through a miRNA gateway miRNAMap 2.0 [44]. Gene annotations were conducted using web-based tools Database for Annotation, Visualization and Integrated Discovery (DAVID, ) [87] or High-throughput GOminer [88]. The significantly (*P *< 0.05) enriched genes involved in biological process for miRNA targets were extracted; and heatmap was created using R 2.6.0.

## Competing interests

The authors declare that they have no competing interests.

## Authors' contributions

JR conducted all experiments for the paper, collected and analyzed the data and wrote the manuscript. PJ carried out data analyses and assisted with writing the manuscript. EW designed the miRNA-array and oligo platform, developed protocol and helped in writing the manuscript. FMM assisted in interpreting the data and provided advice on the manuscript. DFS conceived of the project, provided funding for this work, carried out data analysis and interpretation, and approved the manuscript.

## Supplementary Material

Additional file 1 **The data provided the list of the top20 miRNAs that were differentially expressed among hES cells, embryonic body and adult cells.** Click here for file
