# Supplementary material for: MicroRNA and gene expression patterns in the differentiation of human embryonic stem cells
Source: J Transl Med. 2009 Mar 23;7:20. doi: 10.1186/1479-5876-7-20 (PMC2669448; doi:10.1186/1479-5876-7-20)
Supplement: Additional file 1 — The data provided the list of the top20 miRNAs that were differentially expressed among hES cells, embryonic body and adult cells. [file 1479-5876-7-20-S1.doc]

Additional file 1A. The 20 most upregulated miRNAs in human embryonic stem (hES) cells

| **miRNA** | **hES/Adult ratio** | **hES/EB ratio** | **Expression in hES cells** | **Expression in tissue or tumor** | **Validated Target** | **Function** |
| --- | --- | --- | --- | --- | --- | --- |
| hsa-miR-302b | 57.17 | 1.12 | Undifferentiated [1-4] | heart |  |  |
| hsa-miR-302c | 56.11 | 1.74 | Undifferentiated [1-6] | heart |  |  |
| hsa-mir-302a | 48.49 | 1.39 | Undifferentiated [1-6] | heart |  |  |
| hsa-miR-302d | 45.59 | 1.91 | Undifferentiated [1, 3, 5, 6] | heart |  |  |
| hsa-miR-367 | 26.55 | 2.50 | Undifferentiated [1, 3, 6] |  |  |  |
| hsa-miR-302a* | 17.12 | 1.85 | Undifferentiated [1, 2] |  |  |  |
| hsa-miR-520e | 11.21 | 1.86 | Undifferentiated [1] | placenta |  |  |
| hsa-miR-519e | 6.11 | 3.59 | Undifferentiated [1] | placenta |  |  |
| hsa-miR-520c | 5.91 | 3.21 | Undifferentiated [1] | placenta | CD44 | Promotes breast cancer cells migration and invasion in vitro and in vivo [7] |
| hsa-miR-520b | 5.82 | 4.83 | Undifferentiated [1] | placenta |  |  |
| hsa-miR-520f | 5.80 | 3.06 | Undifferentiated [1] | placenta |  |  |
| hsa-miR-141 | 5.20 | 1.33 |  | epithelial tissues; hematopoietic cells, hepatocellular carcinoma, malignant cholangiocytes, ovarian cancer. | ZEB1 ZEB2  CLOCK | 1. Enhances growth and survival of malignant cholangiocyte in vitro [8].  2. Inhibits mouse carcinoma cell migration and determine epithelial phonotype by increasing E-cadherin expression [9].  3. Inhibits epithelial to mesenchymal transition (EMT) [9-11].  4. Regulates differentiation of zebrafish olfactory progenitor cells [12] |
| hsa-miR-372 | 5.02 | 2.47 | Undifferentiated [1, 2, 6], differentiated (EB) [3] | placenta, testicular germ-cell tumours, squamous Cell Carcinoma | LATS2 | Promotes cell proliferation of testicular germ cell tumor (TGCT) and resistance to oncogenic stress [13] |
| hsa-miR-519c | 4.92 | 4.38 | Undifferentiated [1, 3] | placenta |  |  |
| hsa-miR-515-5p | 4.90 | 3.97 | Undifferentiated [1] | placenta |  |  |
| hsa-miR-519b | 4.60 | 4.47 | Undifferentiated [1] | placenta |  |  |
| hsa-miR-302b* | 4.04 | 1.63 | Undifferentiated [1, 2, 5] |  |  |  |
| hsa-miR-517b | 3.55 | 2.88 | Undifferentiated [1] | placenta |  |  |
| hsa-miR-525* | 3.52 | 2.93 | Undifferentiated [1] | placenta |  |  |
| hsa-miR-200c | 3.41 | 1.05 | Undifferentiated [1, 2, 6] | epithelial tissues of lung, breast, and gastrointestinal organs, hematopoietic cells, hepatocellular carcinoma, malignant cholangiocytes, ovarian cancer, colon cancer | ZEB1 ZEB2  TCF8 | 1.Enhances growth and survival of malignant cholangiocyte in vitro[8].  5. Alters cell morphology of breast cancer, lung cancer by increasing E-cadherin expression [14].  2. Inhibits mouse carcinoma cell migration and determines epithelial phonotype by increasing E-cadherin expression [9].  3. Inhibits epithelial to mesenchymal transition (EMT) [9-11].  4. Regulates differentiation of zebrafish olfactory progenitor cells [12]. |

Additional file 1B. The 20 most upregulated miRNAs in embryonic bodies (EB)

| **miRNA** | **EB/Adult ratio** | **EB/hES ratio** | **Expression in hES cells** | **Expression in tissue or tumor** | **Validated Target** | **Function** |
| --- | --- | --- | --- | --- | --- | --- |
| hsa-miR-20a | 4.77 | 1.58 | differentiated (EB )[3] | ovarian carcinoma, thyroid cancer cells, lung cancer, colon cancer | HIF1A,  E2F1  LRF  TGFBR2 | 1. Regulates cell proliferation and cell survival of lung cance r[15, 16]  2. Decreases cell proliferation and induce cell senescence of mouse embryonic fibroblasts (MEF) [17] |
| hsa-miR-20b | 4.59 | 1.47 | differentiated (EB) [3],  undifferentiated[4] |  | ARID4B | 1. Regulates the G1-S transition of immortal mammary epithelial cells [18]  2. Induces anchorage independence growth of NIH/3Ts cells [19] |
| hsa-miR-17-5p | 4.50 | 1.57 | differentiated (EB)[3, 6],  undifferentiated [1, 2] | B-cell lymphoma, hepatocellular carcinoma, lung cancer, squamous cell carcinoma | E2F1  AIB1 | 1. Regulates the G1-S transition of immortal mammary epithelial cells [18]  2. Induces anchorage independence growth of NIH/3Ts cells [19]  3. Suppresses breast cancer cell proliferation [20]  4. Accelerates tumor development of B-cell lymphoma [21]  4. Regulates development of heart, lung and immune system [22] |
| hsa-miR-190 | 4.26 | 3.64 |  | primary myelofibrosis granulocyte, hepatocellular carcinoma, thyroid cancer |  |  |
| hsa-miR-18a | 4.24 | 1.38 |  | ovarian carcinoma[23], neuronal cells[24], thyroid cancer cells[25] | NR3C1, CTGF | Regulates growth and survival of thyroid cancer cells [25] |
| hsa-miR-106a | 4.01 | 1.55 | differentiated (EB) [3] | thyroid cancer cells, lung cancer, T-cell lymphoma, hepatocellular carcinoma, colon adenocarcinoma, T-leukemia | ARID4B  RB1 | 1. Regulates the G1-S transition of immortal mammary epithelial cells [18]  2. Induces anchorage independence growth of NIH/3Ts cells [19] |
| hsa-miR-93 | 3.90 | 1.48 | differentiated ( EB) [6] | hepatocellular carcinoma, ovarian carcinoma |  | Promotes cell cycle progression [18] |
| hsa-miR-106b | 3.62 | 1.46 | undifferentiated [3] | thyroid cancer cells, lung cancer, hepatocellular carcinoma | CDKN1A | Regulates the G1-S transition [18] |
| hsa-miR-19b | 3.35 | 1.24 | differentiated (EB) [3], undifferentiated [2] | thyroid cancer cells, T-leukemia | ARID4B  MYLIP | Induces anchorage independence growth of NIH/3Ts cells[19] |
| hsa-miR-19a | 3.30 | 1.29 | differentiated (EB )[3], | thyroid cancer cells, lung cancer, | PTEN  TSP1 | 1. Regulates growth and survival of thyroid cancer cells [25]  2. Regulates tumor angiogenesis [26] |
| hsa-miR-219 | 3.19 | 2.54 | differentiated (EB) [3] | brain, hepatocellular carcinoma | SCOP | Fine-tunes the length of circadian period [27] |
| hsa-miR-373 | 3.07 | 1.60 | differentiated (EB) [3],  undifferentiated [1, 2, 6] | placenta, breast cancer, testicular germ-cell tumours | CD44  LATS2 | 1. Involved in tumorigenesis, migration and invasion of cancer cells [7, 13]  2. Induces expression of E-cadherin and CSDC2 by binding their promoter [28] |
| hsa-miR-135a | 3.01 | 1.69 | differentiated (EB) [3] | colorectal carcinomas | APC | Suppresses APC and induces downstream Wnt pathway in colorectal cancer [29] |
| hsa-miR-92 | 2.97 | 1.42 | differentiated (EB) [6] | thyroid cancer cells, T-leukemia | ARID4B  MYLIP  HIPK3 | Induces anchorage independence growth of NIH/3Ts cells [19] |
| hsa-miR-374 | 2.95 | 1.62 |  | HeLa and STO cells, cervical cancer, |  |  |
| hsa-miR-30e-5p | 2.73 | 2.16 | differentiated (EB)[3] | leukemia cells |  |  |
| hsa-miR-101 | 2.62 | 1.22 |  | hepatocellular carcinoma, acute megakaryoblastic leukemia (AMKL) Cell Lines, T-cells | MYCN  EZH2  mTOR  ICOS | Affects human cytomegalovirus replication by targeting mTOR protein [30] |
| hsa-miR-148a | 2.56 | 1.48 | undifferentiated[4] | acute myeloid leukemia, liver | BAALC  PXR | Modulates the inducible and/or constitutive levels of CYP3A4 in human liver [31] |
| hsa-miR-301 | 2.22 | 1.59 | differentiated (EB) [3] | hepatocellular carcinoma, pancreas cancer |  |  |
| hsa-miR-33 | 2.16 | 1.07 | differentiated (EB) [3] | Adult tissues |  |  |

Additional file 1C. The 20 most upregulated miRNAs in adult cells

| **miRNA** | **Adult/hES ratio** | **Adult/EB ratio** | **Expression in hES cells** | **Expression in tissue or tumor** | **Validated target** | **Function** |
| --- | --- | --- | --- | --- | --- | --- |
| hsa-let-7a | 6.13 | 11.03 | undifferentiated [2], differentiated (EB) [3] | lung, breast, brain, embryonic tissue, hepatocellular carcinoma, Hodgkin lymphoma, polycythemia vera granulocytes, gastric cancer, leiomyoma | Ras  HMGA2  MYC  PRDM1  NF2  ITGB3 | 1. Reduces invasive potential of melanoma cells in vivo [32]2. interferse with normal B-cell terminal differentiation [33]  3. Promotes the phosphorylation of Stat-3 and proliferation of malignant human cholangiocytes [34] |
| hsa-let-7f | 4.58 | 8.73 | differentiated (EB) [3] | lung, mesenchymal stem cells (MSC), CD8 T cells, leiomyoma | Ras  HMGA2  KLK6  KLK10 | 1. Tumor suppressor 2. promotes in vitro spout formation [35] |
| hsa-miR-29a | 3.72 | 8.53 |  | brain, ovarian carcinoma, muscle, fat, and liver | BACE1  Abeta | 1. regulates brain development [36]  2. indirectly represses insulin-stimulated glucose uptake [37] |
| hsa-let-7g | 4.38 | 8.34 | Undifferentiated [3] | lung, colon cancer, leiomyoma | Ras  HMGA2 | induces cell cycle arrest and cell death of lung cancer [38] |
| hsa-miR-29b | 2.84 | 7.94 | undifferentiated [2] | muscle, fat, and liver, brain, cholangiocytes | BACE1  Abeta  MCL1 | 1. regulating apoptosis of malignant cholangiocarcinoma cells [39]  2. regulates brain development [36]  3. indirectly repress insulin-stimulated glucose uptake [37] |
| hsa-let-7c | 4.31 | 6.28 | undifferentiated [3] | lung, gastric cancer, brain, leiomyoma | Ras HMGA2  c-myc | Tumor suppressor |
| hsa-miR-29c | 2.90 | 6.21 | differentiated (EB) [3] | mesenchymal stem cells (MSC), muscle, fat, and liver, nasopharyngeal carcinomas | collagen 4A1, 15A1, LAMC1  TDG | 1. involved in nasopharyngeal carcinoma metastasis [40]  2. indirectly represses insulin-stimulated glucose uptake [37] |
| hsa-let-7b | 4.00 | 5.99 |  | lung, gastric cancer, melanoma, ovarian carcinoma, leiomyoma, melanoma | Ras HMGA2  CCND1  CCND3  CCNA2  CDK4  LIN28  MTPN | Tumor suppressor |
| hsa-let-7i | 4.55 | 5.84 | undifferentiated [3] | lung, leiomyoma, cholangiocytes | Ras HMGA2  TLR4 | 1.Tumor suppressor  2. regulates cholangiocyte immune response against Cryptosporidum parvum infection [41] |
| hsa-miR-99a | 6.86 | 5.18 | differentiated (EB) [3] | acute megakaryoblastic leukemia (AMKL) Cell Lines, ovarian cancer and squamous cell carcinoma |  |  |
| hsa-miR-368 | 3.08 | 4.82 | undifferentiated [2] | Acute myeloid leukemia |  |  |
| hsa-miR-31 | 3.14 | 4.55 | undifferentiated [3] | epithelial tissues, squamous cell carcinoma, hepatocellular carcinoma, colorectal cancer |  |  |
| hsa-let-7e | 2.97 | 4.49 | differentiated (EB )[3, 4] | lung, leiomyoma, leukemia cells | Ras HMGA2  SMC1A | Tumor suppressor |
| hsa-let-7d | 3.00 | 3.65 | undifferentiated [3] | lung, leiomyoma, acute promyelocytic leukemia cells | Ras HMGA2 | Tumor suppressor |
| hsa-miR-155 | 2.26 | 3.47 | undifferentiated [3] | hematopoietic cells, CD8 T cells, B-cell lymphoma, squamous cell carcinoma, pancreatic cancer, Acute myeloid leukaemia, cervical cancer, thyroid tumors | SPI1  AGTR1 | 1.Regulates immune function by physiological granulocyte/monocyte expansion [42] or production and memory response of B-cells [43]  2. Its abnormal expression is associated with AML [42, 43]  3. Reduces the expression of the AGTR1 and ERK1/2 activation in human primary lung fibroblasts [44] |
| hsa-miR-221 | 2.79 | 3.44 | undifferentiated [3] | neuronal progenitor cells, brain, thyroid carcinomas, pancreatic cancer, acute myeloid leukaemia, hepatocellular carcinoma, ovarian cancer | KIT  CDKN1B CDKN1C | 1. Inhibit normal erythropoiesis and erythroleukemic cell growth [45]  2. Promotes growth of tumor cells by increasing the number of cells in S-phase [46-48]  3. Anti-angiogenic effects [49] |
| hsa-miR-495 | 2.22 | 3.10 | differentiated (EB) [3] |  |  |  |
| hsa-miR-21 | 2.21 | 3.10 | undifferentiated [2, 3, 6] | CD8 T cells, AML, breast cancer, colorectal cancer, hepatocellular carcinoma, ovarian carcinoma, squamous cell carcinoma, pancreatic cancer, endothelial cells, glioblastoma, | RECK TIMP3  PDCD4 PTEN TPM1 | Promotes cell proliferation, migration, and invasion [50-53] |
| hsa-miR-222 | 2.50 | 2.95 | undifferentiated [2, 3, 6] | CD8 T cells, AML, breast cancer, colorectal cancer, hepatocellular carcinoma, ovarian carcinoma, squamous cell carcinoma, pancreatic cancer, endothelial cells, glioblastoma, | KIT  CDKN1BCDKN1C | 1. Inhibits normal erythropoiesis and erythroleukemic cell growth [45]  2. Promotes growth of tumor cells by increasing the number of cells in S-phase [46-48]  3. Anti-angiogenic effects [49] |
| hsa-miR-100 | 5.92 | 2.92 |  | ovarian carcinoma, squamous cell carcinoma, endothelial cells | mTOR  raptor | over-expression  of miR-100 is involved in  the beta-adrenergic  receptor-mediated  repression of cardiac  genes [54] |

1. Laurent LC, Chen J, Ulitsky I, Mueller FJ, Lu C, Shamir R, Fan JB, Loring JF: **Comprehensive microRNA profiling reveals a unique human embryonic stem cell signature dominated by a single seed sequence**. *Stem Cells* 2008, **26**(6):1506-1516.

2. Suh MR, Lee Y, Kim JY, Kim SK, Moon SH, Lee JY, Cha KY, Chung HM, Yoon HS, Moon SY *et al*: **Human embryonic stem cells express a unique set of microRNAs**. *Dev Biol* 2004, **270**(2):488-498.

3. Morin RD, O'Connor MD, Griffith M, Kuchenbauer F, Delaney A, Prabhu AL, Zhao Y, McDonald H, Zeng T, Hirst M *et al*: **Application of massively parallel sequencing to microRNA profiling and discovery in human embryonic stem cells**. *Genome Res* 2008, **18**(4):610-621.

4. Bar M, Wyman SK, Fritz BR, Qi J, Garg KS, Parkin RK, Kroh EM, Bendoraite A, Mitchell PS, Nelson AM *et al*: **MicroRNA Discovery and Profiling in Human Embryonic Stem Cells by Deep Sequencing of Small RNA Libraries**. *Stem Cells* 2008.

5. Wu H, Xu J, Pang ZP, Ge W, Kim KJ, Blanchi B, Chen C, Sudhof TC, Sun YE: **Integrative genomic and functional analyses reveal neuronal subtype differentiation bias in human embryonic stem cell lines**. *Proc Natl Acad Sci U S A* 2007, **104**(34):13821-13826.

6. Lakshmipathy U, Love B, Goff LA, Jornsten R, Graichen R, Hart RP, Chesnut JD: **MicroRNA expression pattern of undifferentiated and differentiated human embryonic stem cells**. *Stem Cells Dev* 2007, **16**(6):1003-1016.

7. Huang Q, Gumireddy K, Schrier M, le Sage C, Nagel R, Nair S, Egan DA, Li A, Huang G, Klein-Szanto AJ *et al*: **The microRNAs miR-373 and miR-520c promote tumour invasion and metastasis**. *Nat Cell Biol* 2008, **10**(2):202-210.

8. Meng F, Henson R, Lang M, Wehbe H, Maheshwari S, Mendell JT, Jiang J, Schmittgen TD, Patel T: **Involvement of human micro-RNA in growth and response to chemotherapy in human cholangiocarcinoma cell lines**. *Gastroenterology* 2006, **130**(7):2113-2129.

9. Korpal M, Lee ES, Hu G, Kang Y: **The miR-200 family inhibits epithelial-mesenchymal transition and cancer cell migration by direct targeting of E-cadherin transcriptional repressors ZEB1 and ZEB2**. *J Biol Chem* 2008, **283**(22):14910-14914.

10. Gregory PA, Bert AG, Paterson EL, Barry SC, Tsykin A, Farshid G, Vadas MA, Khew-Goodall Y, Goodall GJ: **The miR-200 family and miR-205 regulate epithelial to mesenchymal transition by targeting ZEB1 and SIP1**. *Nat Cell Biol* 2008, **10**(5):593-601.

11. Park SM, Gaur AB, Lengyel E, Peter ME: **The miR-200 family determines the epithelial phenotype of cancer cells by targeting the E-cadherin repressors ZEB1 and ZEB2**. *Genes Dev* 2008, **22**(7):894-907.

12. Choi PS, Zakhary L, Choi WY, Caron S, Alvarez-Saavedra E, Miska EA, McManus M, Harfe B, Giraldez AJ, Horvitz RH *et al*: **Members of the miRNA-200 family regulate olfactory neurogenesis**. *Neuron* 2008, **57**(1):41-55.

13. Voorhoeve PM, le Sage C, Schrier M, Gillis AJ, Stoop H, Nagel R, Liu YP, van Duijse J, Drost J, Griekspoor A *et al*: **A genetic screen implicates miRNA-372 and miRNA-373 as oncogenes in testicular germ cell tumors**. *Cell* 2006, **124**(6):1169-1181.

14. Hurteau GJ, Carlson JA, Spivack SD, Brock GJ: **Overexpression of the microRNA hsa-miR-200c leads to reduced expression of transcription factor 8 and increased expression of E-cadherin**. *Cancer Res* 2007, **67**(17):7972-7976.

15. Matsubara H, Takeuchi T, Nishikawa E, Yanagisawa K, Hayashita Y, Ebi H, Yamada H, Suzuki M, Nagino M, Nimura Y *et al*: **Apoptosis induction by antisense oligonucleotides against miR-17-5p and miR-20a in lung cancers overexpressing miR-17-92**. *Oncogene* 2007, **26**(41):6099-6105.

16. Taguchi A, Yanagisawa K, Tanaka M, Cao K, Matsuyama Y, Goto H, Takahashi T: **Identification of hypoxia-inducible factor-1 alpha as a novel target for miR-17-92 microRNA cluster**. *Cancer Res* 2008, **68**(14):5540-5545.

17. Poliseno L, Pitto L, Simili M, Mariani L, Riccardi L, Ciucci A, Rizzo M, Evangelista M, Mercatanti A, Pandolfi PP *et al*: **The proto-oncogene LRF is under post-transcriptional control of MiR-20a: implications for senescence**. *PLoS ONE* 2008, **3**(7):e2542.

18. Ivanovska I, Ball AS, Diaz RL, Magnus JF, Kibukawa M, Schelter JM, Kobayashi SV, Lim L, Burchard J, Jackson AL *et al*: **MicroRNAs in the miR-106b family regulate p21/CDKN1A and promote cell cycle progression**. *Mol Cell Biol* 2008, **28**(7):2167-2174.

19. Landais S, Landry S, Legault P, Rassart E: **Oncogenic potential of the miR-106-363 cluster and its implication in human T-cell leukemia**. *Cancer Res* 2007, **67**(12):5699-5707.

20. Hossain A, Kuo MT, Saunders GF: **Mir-17-5p regulates breast cancer cell proliferation by inhibiting translation of AIB1 mRNA**. *Mol Cell Biol* 2006, **26**(21):8191-8201.

21. Sempere LF, Christensen M, Silahtaroglu A, Bak M, Heath CV, Schwartz G, Wells W, Kauppinen S, Cole CN: **Altered MicroRNA expression confined to specific epithelial cell subpopulations in breast cancer**. *Cancer Res* 2007, **67**(24):11612-11620.

22. Ventura A, Young AG, Winslow MM, Lintault L, Meissner A, Erkeland SJ, Newman J, Bronson RT, Crowley D, Stone JR *et al*: **Targeted deletion reveals essential and overlapping functions of the miR-17 through 92 family of miRNA clusters**. *Cell* 2008, **132**(5):875-886.

23. Nam EJ, Yoon H, Kim SW, Kim H, Kim YT, Kim JH, Kim JW, Kim S: **MicroRNA expression profiles in serous ovarian carcinoma**. *Clin Cancer Res* 2008, **14**(9):2690-2695.

24. Uchida S, Nishida A, Hara K, Kamemoto T, Suetsugi M, Fujimoto M, Watanuki T, Wakabayashi Y, Otsuki K, McEwen BS *et al*: **Characterization of the vulnerability to repeated stress in Fischer 344 rats: possible involvement of microRNA-mediated down-regulation of the glucocorticoid receptor**. *Eur J Neurosci* 2008, **27**(9):2250-2261.

25. Takakura S, Mitsutake N, Nakashima M, Namba H, Saenko VA, Rogounovitch TI, Nakazawa Y, Hayashi T, Ohtsuru A, Yamashita S: **Oncogenic role of miR-17-92 cluster in anaplastic thyroid cancer cells**. *Cancer Sci* 2008, **99**(6):1147-1154.

26. Dews M, Homayouni A, Yu D, Murphy D, Sevignani C, Wentzel E, Furth EE, Lee WM, Enders GH, Mendell JT *et al*: **Augmentation of tumor angiogenesis by a Myc-activated microRNA cluster**. *Nat Genet* 2006, **38**(9):1060-1065.

27. Cheng HY, Papp JW, Varlamova O, Dziema H, Russell B, Curfman JP, Nakazawa T, Shimizu K, Okamura H, Impey S *et al*: **microRNA modulation of circadian-clock period and entrainment**. *Neuron* 2007, **54**(5):813-829.

28. Place RF, Li LC, Pookot D, Noonan EJ, Dahiya R: **MicroRNA-373 induces expression of genes with complementary promoter sequences**. *Proc Natl Acad Sci U S A* 2008, **105**(5):1608-1613.

29. Nagel R, le Sage C, Diosdado B, van der Waal M, Vrielink JA, Bolijn A, Meijer GA, Agami R: **Regulation of the adenomatous polyposis coli gene by the miR-135 family in colorectal cancer**. *Cancer Res* 2008, **68**(14):5795-5802.

30. Wang FZ, Weber F, Croce C, Liu CG, Liao X, Pellett PE: **Human cytomegalovirus infection alters expression of cellular microRNA species that affect its replication**. *J Virol* 2008.

31. Takagi S, Nakajima M, Mohri T, Yokoi T: **Post-transcriptional regulation of human pregnane X receptor by micro-RNA affects the expression of cytochrome P450 3A4**. *J Biol Chem* 2008, **283**(15):9674-9680.

32. Muller DW, Bosserhoff AK: **Integrin beta(3) expression is regulated by let-7a miRNA in malignant melanoma**. *Oncogene* 2008.

33. Nie K, Gomez M, Landgraf P, Garcia JF, Liu Y, Tan LH, Chadburn A, Tuschl T, Knowles DM, Tam W: **MicroRNA-mediated down-regulation of PRDM1/Blimp-1 in Hodgkin/Reed-Sternberg cells: a potential pathogenetic lesion in Hodgkin lymphomas**. *Am J Pathol* 2008, **173**(1):242-252.

34. Meng F, Henson R, Wehbe-Janek H, Smith H, Ueno Y, Patel T: **The MicroRNA let-7a modulates interleukin-6-dependent STAT-3 survival signaling in malignant human cholangiocytes**. *J Biol Chem* 2007, **282**(11):8256-8264.

35. Kuehbacher A, Urbich C, Zeiher AM, Dimmeler S: **Role of Dicer and Drosha for endothelial microRNA expression and angiogenesis**. *Circ Res* 2007, **101**(1):59-68.

36. Hebert SS, Horre K, Nicolai L, Papadopoulou AS, Mandemakers W, Silahtaroglu AN, Kauppinen S, Delacourte A, De Strooper B: **Loss of microRNA cluster miR-29a/b-1 in sporadic Alzheimer's disease correlates with increased BACE1/beta-secretase expression**. *Proc Natl Acad Sci U S A* 2008, **105**(17):6415-6420.

37. He A, Zhu L, Gupta N, Chang Y, Fang F: **Overexpression of micro ribonucleic acid 29, highly up-regulated in diabetic rats, leads to insulin resistance in 3T3-L1 adipocytes**. *Mol Endocrinol* 2007, **21**(11):2785-2794.

38. Kumar MS, Erkeland SJ, Pester RE, Chen CY, Ebert MS, Sharp PA, Jacks T: **Suppression of non-small cell lung tumor development by the let-7 microRNA family**. *Proc Natl Acad Sci U S A* 2008, **105**(10):3903-3908.

39. Mott JL, Kobayashi S, Bronk SF, Gores GJ: **mir-29 regulates Mcl-1 protein expression and apoptosis**. *Oncogene* 2007, **26**(42):6133-6140.

40. Sengupta S, den Boon JA, Chen IH, Newton MA, Stanhope SA, Cheng YJ, Chen CJ, Hildesheim A, Sugden B, Ahlquist P: **MicroRNA 29c is down-regulated in nasopharyngeal carcinomas, up-regulating mRNAs encoding extracellular matrix proteins**. *Proc Natl Acad Sci U S A* 2008, **105**(15):5874-5878.

41. Chen XM, Splinter PL, O'Hara SP, LaRusso NF: **A cellular micro-RNA, let-7i, regulates Toll-like receptor 4 expression and contributes to cholangiocyte immune responses against Cryptosporidium parvum infection**. *J Biol Chem* 2007, **282**(39):28929-28938.

42. O'Connell RM, Rao DS, Chaudhuri AA, Boldin MP, Taganov KD, Nicoll J, Paquette RL, Baltimore D: **Sustained expression of microRNA-155 in hematopoietic stem cells causes a myeloproliferative disorder**. *J Exp Med* 2008, **205**(3):585-594.

43. Vigorito E, Perks KL, Abreu-Goodger C, Bunting S, Xiang Z, Kohlhaas S, Das PP, Miska EA, Rodriguez A, Bradley A *et al*: **microRNA-155 regulates the generation of immunoglobulin class-switched plasma cells**. *Immunity* 2007, **27**(6):847-859.

44. Martin MM, Lee EJ, Buckenberger JA, Schmittgen TD, Elton TS: **MicroRNA-155 regulates human angiotensin II type 1 receptor expression in fibroblasts**. *J Biol Chem* 2006, **281**(27):18277-18284.

45. Felli N, Fontana L, Pelosi E, Botta R, Bonci D, Facchiano F, Liuzzi F, Lulli V, Morsilli O, Santoro S *et al*: **MicroRNAs 221 and 222 inhibit normal erythropoiesis and erythroleukemic cell growth via kit receptor down-modulation**. *Proc Natl Acad Sci U S A* 2005, **102**(50):18081-18086.

46. Fornari F, Gramantieri L, Ferracin M, Veronese A, Sabbioni S, Calin GA, Grazi GL, Giovannini C, Croce CM, Bolondi L *et al*: **MiR-221 controls CDKN1C/p57 and CDKN1B/p27 expression in human hepatocellular carcinoma**. *Oncogene* 2008.

47. Medina R, Zaidi SK, Liu CG, Stein JL, van Wijnen AJ, Croce CM, Stein GS: **MicroRNAs 221 and 222 bypass quiescence and compromise cell survival**. *Cancer Res* 2008, **68**(8):2773-2780.

48. Felicetti F, Errico MC, Bottero L, Segnalini P, Stoppacciaro A, Biffoni M, Felli N, Mattia G, Petrini M, Colombo MP *et al*: **The promyelocytic leukemia zinc finger-microRNA-221/-222 pathway controls melanoma progression through multiple oncogenic mechanisms**. *Cancer Res* 2008, **68**(8):2745-2754.

49. Poliseno L, Tuccoli A, Mariani L, Evangelista M, Citti L, Woods K, Mercatanti A, Hammond S, Rainaldi G: **MicroRNAs modulate the angiogenic properties of HUVECs**. *Blood* 2006, **108**(9):3068-3071.

50. Gabriely G, Wurdinger T, Kesari S, Esau CC, Burchard J, Linsley PS, Krichevsky AM: **MiR-21 Promotes Glioma Invasion by Targeting MMP Regulators**. *Mol Cell Biol* 2008.

51. Asangani IA, Rasheed SA, Nikolova DA, Leupold JH, Colburn NH, Post S, Allgayer H: **MicroRNA-21 (miR-21) post-transcriptionally downregulates tumor suppressor Pdcd4 and stimulates invasion, intravasation and metastasis in colorectal cancer**. *Oncogene* 2008, **27**(15):2128-2136.

52. Meng F, Henson R, Wehbe-Janek H, Ghoshal K, Jacob ST, Patel T: **MicroRNA-21 regulates expression of the PTEN tumor suppressor gene in human hepatocellular cancer**. *Gastroenterology* 2007, **133**(2):647-658.

53. Zhu S, Si ML, Wu H, Mo YY: **MicroRNA-21 targets the tumor suppressor gene tropomyosin 1 (TPM1)**. *J Biol Chem* 2007, **282**(19):14328-14336.

54. Sucharov C, Bristow MR, Port JD: **miRNA expression in the failing human heart: functional correlates**. *J Mol Cell Cardiol* 2008, **45**(2):185-192.
